# The data of *Escherichia coli* strains genes in different types of wastewater

**DOI:** 10.1016/j.dib.2018.08.167

**Published:** 2018-08-31

**Authors:** Mojtaba Afsharnia, Behnaz Naraghi, Jalal Mardaneh, Mojtaba Kianmehr, Hamed Biglari

**Affiliations:** a Department of Environmental Health Engineering, School of Public Health, Social Development & Health Promotion Research Center, Gonabad University of Medical Sciences, Gonabad, Iran; b Microbiology Department, School of Medicine, Gonabad University of Medical Sciences, Gonabad, Iran; c Department of Medical Physics, Faculty of Medicine, Gonabad University of Medical Sciences, Gonabad, Iran

**Keywords:** *Escherichia coli*, Wastewater, Virulence genes, Resistance genes, Antibiotic resistance

## Abstract

From April 2016 to March 2017, a number of 99 isolates of *Escherichia coli* were collected from three types of wastewater including urban wastewater (33 isolates), livestock slaughterhouse wastewater (33 isolates) and poultry slaughterhouse wastewater (33 isolate). The specimens were cultured on microbiological media. The bacterial identification was performed by morphological and biochemical tests. Polymerase chain reaction (PCR) method was carried out to detect 2 virulence genes (*traT*, and *fimH*) and 4 antibiotic resistance genes (*bla*_*TEM, CTX, SHV*_, and *tetA*). The data showed that the prevalence rate of *traT, fimH,blaCTX, blaTEM,blaSHV, tetA* genes were 89.9%, 91.9%, 79.8%, 40.4%, 6.1%, and 91.9%, respectively.

**Specifications table**

**Table:** 

Subject area	Environmental Sciences
More specific subject area	Microbiology
Type of data	Tables and Figures
How data was acquired	From April 2016 to March 2017, a total of 99 non-duplicate isolates of *Escherichia coli* were recovered from three type sewage including poultry wastewater (33 isolates), urban sewage (33 isolates), and livestock slaughterhouse wastewater (33 isolates) in Gonabad, northeast of Iran. The specimens were cultured on microbiological media. All bacterial isolates identified and confirmed as *Escherichia coli* by morphological and biochemical tests. The bacterial cells were cultured overnight on Mueller-Hinton agar. The whole genomic DNA was extracted from single colonies using boiling method and used as a template for PCR amplification
Data format	Raw, analyzed
Experimental factors	Prototype strain *Escherichia coli* ATCC 25922 was applied as quality control strain throughout this research. DNA ladder (50 bp size range) was used to detect the *size* of the expected bands.
Experimental features	Whole genomic DNA was extracted from single colonies using boiling method and used as a template for PCR amplification.
Data source location	Gonabad County, Khorasan Razavi Province, Iran
Data accessibility	Data are included in this article

**Value of the data**

• The data can be useful to operators of water and wastewater treatment plants for better microbial contamination control and the need to be aware of the prevalent amount of pathogenic *E. coli* genes.
• The data can be used to show that the prevalence of pathogenic *E. coli* genes in different environment of urban wastewater, livestock slaughterhouse wastewater and poultry slaughterhouse wastewater are various and treatment of them must be done by different methods.
• The gene of *E. coli* strain isolated from urban wastewater, livestock slaughterhouse wastewater and poultry slaughterhouse wastewater are different, and the prevalence of fimH and tetA genes were much higher than other genes in three types of wastewater.
• The data can be used to show that high prevalence of virulence traits was observed in urban wastewater and need to be considered as a health-alarming situation.
• The prevalence of antibiotic resistance genes of pathogenic *E. coli* in urban wastewater was much higher than *E.coli* bacteria present in livestock slaughterhouse wastewater and poultry slaughterhouse wastewater, respectively.

## 1 Data

The prevalence rate of traT, fimH, blaCTX, blaTEM, blaSHV, and tetA genes in poultry slaughterhouse wastewater isolate bacteria were 81.8%, 84.8%, 72.7%, 45.5%, 3%, and 87.9% respectively. The prevalence rate of traT, fimH, blaCTX, blaTEM, blaSHV, and tetA genes in urban wastewater isolate bacteria were 93.9%, 93.9%, 87.9%, 39.4%, 6.1%, and 100%, respectively. The prevalence rate of traT, fimH, blaCTX, blaTEM, blaSHV, and tetA genes in livestock slaughterhouse wastewater isolate bacteria were 93.9%, 97%, 78.8%, 36.4%, 9.1% and 87.9%, respectively, see Table 1. The prevalence rate of traT gene in isolates from urban wastewater and livestock slaughterhouse wastewater was 93.9% and the prevalence rate of fimH gene in the isolates from urban wastewater and livestock slaughterhouse wastewater were 93.9% and 97%, respectively.The prevalence of resistance gene was belonged to tetA gene. The SHV gene has the least prevalence among all isolates.

**Table 1:** Frequency of studied genes among strains isolated from different wastewater sources.

Source	Virulence genes %		Resistance genes %			
	*traT*	*fimH*	*blaCTX*	*blaTEM*	*blaSHV*	*tetA*
Poultry sewage (number = 33)	27	28	24	15	1	29
	81.8%	84.8%	72.7%	45.5%	3%	87.9%
Urban sewage (number = 33)	31	31	29	13	2	33
	93.9%	93.9%	87.9%	39.4%	6.1%	100%
Livestock slaughter house sewage (number = 33)	31	32	26	12	3	29
	93.9%	97%	78.8%	36.4%	9.1%	87.9%
Total (number = 99)	89	91	79	40	6	91
	89.9%	91.9%	79.8%	40.4%	6.1%	91.9%

## 2 Experimental design, materials and methods

### 2.1 Sample collection

For the prepared the dataset of this article from April 2016 to March 2017, a number of 99 non-duplicate isolates of *Escherichia coli* were recovered from three types of wastewater including poultry slaughterhouse wastewater (33 isolates), urban wastewater (33 isolates), and livestock slaughterhouse wastewater (33 isolate) located in Gonabad, Iran.

### 2.2 Bacterial identification

Wastewater samples (250 ml) were collected aseptically in sterile glass bottles [1], [2]. Specimens were sent to the clinical microbiology laboratory within 1 h of specimen collection. The collected specimens were cultured on MacConkey and incubated for 24 h at 35 °C ± 2 [3], [4]. Primary bacterial identification was performed by standard diagnostic tests. Briefly, the overnight pure growth of the organisms on MacConkey agar plates was checked on the basis of Gram staining,colonial morphology and lactose fermentation [5], [6]. The isolated colonies were final identified by oxidase, catalase, motility, triple sugar iron agar (TSI) inoculation, citrate utilization, indole, and H_2_S production. The pure bacterial colonies were inoculated onto medium containing 1.5 ml of sterile Tryptic Soy broth (TSB) mixed with glycerol (20%) and stored at −20 °C for further investigation [7,8].

### 2.3 Detection of virulence and resistance genes by polymerase chain reaction (PCR)

For detection of virulence and resistance genes at first the bacterial cells were culture overnight on Mueller-Hinton agar. After than boiling and the PCR methods were used for detection and distribution of virulence genes and antibiotic resistance genes *E. coli* isolates, Table 2 [9], [10].

**Table 2:** Nucleotide sequences of primers and conditions used to amplify species specific, virulence markers and antibiotic resistance genes in *E.coli* isolates by PCR.

**Virulence factor**	**Target gene**	**Primer name**	**Sequence (5′ to 3′)**	**Length (bp)**	**Annealing temperature (**°C**)**	**Amplicon size (pb)**	**References**
*traT*	***traT***	*traT-F*	GGTGTGGTGCGATGAGCACAG	21	60	290	[11]
		*traT-R*	CACGGTTCAGCCATCCCTGAG	21			
*fimH*	***fimH***	*fimH-F*	CATTCGCCTGTAAAACCGCC	20	60	207	[12]
		*fimH-R*	ATAACACGCCGCCATAAGCC	20			
*blaCTX*	***blaCTX***	*blaCTX-F*	TTTGCGATGTGCAGTACCAGTAA	23	55	544	[12]
		*blaCTX-R*	CGATATCGTTGGTGGTGCCATA	23			
*tetA*	***tetA***	*tetA-F*	TTGGCATTCTGCATTCACTC	20	60	494	[9]
		*tetA-R*	GTATAGCTTGCCGGAAGTCG	20			
*blaTEM*	***blaTEM***	*TEM-F*	ATAAAATTCTTGAAGACGAAA	19	50	1150	[13]
		*TEM-R*	GACAGTTACCAATGCTTAATCA	19			
*blaSHV*	***blaSHV***	*SHV-F*	CACTCAAGGATGTATTGTG	19	50	885	[10]
		*SHV-R*	TTAGCGTTGCCAGTGCTCG	19			
