# Supplementary material for: The data of Escherichia coli strains genes in different types of wastewater
Source: Data Brief. 2018 Aug 31;21:763–6. doi: 10.1016/j.dib.2018.08.167 (PMC6216088; doi:10.1016/j.dib.2018.08.167)
Supplement: Supplementary file 1 — Supplementary material [file mmc1.docx]

Conflict of Interest

All the authors confirm as No conflict of Interest
